# Effects of Different Selenium Sources on Meat Quality and Shelf Life of Fattening Pigs

**DOI:** 10.3390/ani10040615

**Published:** 2020-04-03

**Authors:** Shaotao Zhang, Yuhuai Xie, Min Li, Haitao Yang, Shiyin Li, Junhui Li, Qingqing Xu, Weiren Yang, Shuzhen Jiang

**Affiliations:** Shandong Provincial Key Laboratory of Animal Biotechnology and Disease Control and Prevention, College of Animal Science and Veterinary Medicine, Shandong Agricultural University, Tai’an 271018, Shandong, China; 15662063565@163.com (S.Z.); yuhuai12@163.com (Y.X.); jay285600@163.com (M.L.); yht8290168@163.com (H.Y.); lishiyin5251@163.com (S.L.); ljh2018110389@163.com (J.L.); xqq0413@163.com (Q.X.)

**Keywords:** organic Se, sodium selenite, meat quality, shelf life

## Abstract

**Simple Summary:**

This study was conducted to assess the effects of different Se sources on the growth performance, carcass performance, meat quality and shelf life of fattening pigs. A control diet was supplemented with SS, and experimental diets were supplemented SY, Se-Met, and SS + Se-Met, respectively. The data showed that using organic Se in fattening pig’s diet could improve meat quality and prolong the shelf life of pork. Thus, replacing inorganic Se in diet with organic Se improved meat quality and pork shelf life of fattening pigs significantly under the conditions of the current study.

**Abstract:**

The aim of this study was to evaluate the effects of different Se sources on the meat quality and shelf life of fattening pigs. The control diet was supplemented with 0.3 mg/kg of Se from sodium selenite (SS), and experimental diets included 0.3, 0.3 and 0.15 + 0.15 mg/kg of Se from Se-enriched yeast (SY), selenomethionine (Se-Met) and SS + Se-Met, respectively. The results showed that using organic Se or Se + Se-Met in fattening pigs’ diet could increase average daily gain (ADG) (*p* < 0.05), decrease F/G (*p* < 0.05), reduce (*p* < 0.01) moisture, drip loss and cooking loss of longissimus thoracis, as well as increase (*p* < 0.05) protein and fat contents of longissimus thoracis. Diet supplementation with SY or Se + Se-Met could increase (*p* < 0.01) back fat thickness and skin thickness, and SY could increase (*p* < 0.01) belly fat rat. Adding SY or Se + Se-Met could reduce (*p* < 0.01) *L* value (45 min, 24 h). Adding Se-Met could decrease (*p* < 0.01) b value (45 min, 24 h), adding Se + Se-Met could reduce b value (45 min), and adding SY could reduce the b value (24 h). However, there were no (*p* < 0.05) significant effects on dressing percentage, carcass sloping length, eye muscle area, pH, a value (45 min) and a value (24 h) of longissimus thoracis. Moreover, the TVB-N contents of longissimus thoracis on the first and fifth days, the numbers of *Lactobacillus* on the third to seventh days and the numbers of *E. coli* in in the fifth to seventh days of longissimus thoracis were reduced (*p* < 0.01) by diet supplementation with organic Se. In conclusion, all the results indicate that replacing inorganic Se in diet with organic Se could improve meat quality of fattening pigs. In addition, organic Se could reduce the total volatile basic nitrogen (TVB-N) contents of longissimus thoracis and reduce the numbers of *E. coli* and *Lactobacillus* in longissimus thoracis, prolonging the shelf life of pork. These results demonstrated that organic Se supplementation was more effective than SS supplementation for meat quality and the shelf life of fattening pigs.

## 1. Introduction

Selenium (Se), an important trace mineral, is essential for animal nutrition. It is the component of at least 25 selenoproteins that participate in redox balance maintenance and antioxidant defences [1]. Adding appropriate levels of inorganic Se (such as sodium selenite (SS)) to the diet can promote the growth and development of animals, but excessive intake of Se will cause some negative effects, such as lower intake, growth inhibition, hair loss, hoof loss, cirrhosis and anemia [2,3]. Compared with traditional inorganic Se sources, organic Se sources (such as selenomethionine (Se-Met), and Se-enriched yeast (SY)) had lower toxicity, higher bioavailability and lower environmental pollution in animal production and application [4,5,6]. In addition, SY is the organic Se enriched in the protein structure of growing yeast cells, mainly in the form of Se-Met (75%), in addition, there are also Se organic compounds such as selenocysteine and methyl selenocysteine [7,8]. Numerous experimental studies have since established that Se-Met and SY are suitable for nutritional Se supplementation [9,10,11].

Drip loss, pH and meat color are regarded as the most important meat quality characteristics [12]. It has been reported that organic Se is superior to inorganic Se in reducing water loss, improving meat color and pH [13,14,15,16]. The content of total volatile basic nitrogen (TVB-N) and the number of microbes are important factors affecting the shelf life of longissimus thoracis. It has been reported that lipid peroxidation induced by free radicals could attack polyunsaturated fatty acids in muscle, which is one of the most important causes of meat deterioration during cold storage. The glutathione peroxidase (GSH-Px) family plays an important role in the body’s antioxidant system. Se can bind to the active center of GSH-Px, which plays a significant role in scavenging free radicals *in vivo* and protecting the integrity of cell membranes [17,18]. At the current stage, few studies had been conducted to explore the effect of Se on the shelf life of meat as well as its mechanisms. Therefore, the objectives of the current study were to evaluate the effects of different Se sources on the growth performance, meat quality, and gross composition of longissimus thoracis and shelf life of fattening pigs.

## 2. Material and Methods

### 2.1. Experimental Design and Feeding Management

A 50-d feeding experiment was conducted at a commercial operation (Huayu piggery, Dongying, China). All protocols used were consistent with the Guide for the Care and Use of Laboratory Animals and approved by the Committee on the Ethics (Approval Number: S20180058) of Shandong Agricultural University (Tai’an, China).

One hundred and sixty 110-d-old crossbred (Duroc × Landrace × Large White) pigs with body weight (BW) of 62.06 ± 0.54 (mean ± SD) kg were allocated to 16 pens (5 barrows and 5 gilts/pen), and the pens were assigned randomly to 4 treatments with 4 pens per treatment. There was no difference in initial average body weight between treatments. The corn-soybean meal-based basal diet (Table 1), analyzed to contain 0.043 mg/kg of Se, was formulated to meet or exceed all nutritional requirements for finishing pigs (60–105 kg, NRC 2012), except for Se. The control group was supplemented with 0.3 mg/kg Se derived from sodium selenite (SS) on the basal diet. The experimental treatments were supplemented with 0.3 mg/kg Se from Se-enriched yeast (SY), selenomethionine (Se-Met), and SY + Se-Met (0.15 + 0.15 mg/kg) on the basal diet, respectively.

The feed-grade SS (Se content is 0.20%), SY (Se content is 0.20%) and *L*-Se-Met (Se content is 0.20%) were obtained from Sichuan Xinyimei Biotechnology Co., Ltd. (Mianyang, China).

The experiment was conducted in Huayu Pig Breeding Farm of Guangrao, Dongying, Shandong Province, China. The experimental pigs were fed in the same pigsty. Preparations for cleaning and disinfection of pigsty were made before the start of the experiment. At the beginning of the experiment, the pigs were fed at eight a.m. daily, and the feed volume was guaranteed to ensure that there was a small amount of leftover material in the trough on the second day. Piggery hygiene, pig immunization, insect repellent and other feeding managements were performed strictly according to the routine procedures of the piggery. Pigs were fed daily for ad libitum intake and had free access to water throughout the whole experiment period. 

### 2.2. Determination of ADG, ADFI and F/G

Pigs were weighed individually on day 0 (110-d-old) and day 50 (160-d-old) before feeding during the experiment to determine the average daily gain (ADG). Feed intakes, orts and spillages were collected and weighed every day to determine the average daily feed intake (ADFI). The feed efficiency was calculated according to the feed gain ratio (F/G).

### 2.3. Determination of Carcass Performance and Meat Quality

After the carcass was splitted, both sides of the carcass (including belly fat and kidney), belly fat, feet, and head were weighed and recorded. Carcass length was recorded by determining the distance between the midpoint of the anterior margin of pubic symphysis and the midpoint of the anterior margin of the first cervical spine of the left side of the carcass. The carcass sloping length was recorded by determining the distance between the midpoint of the anterior margin of pubic symphysis and the junction of the first rib and sternum of the left side of the carcass. Back fat thickness and skin thickness were measured at the 6th-7th thoracic vertebral junction perpendicular to the back on the left side of the carcass. Both sides of the belly fat were weighed and the belly fat rate was calculated using the following formula:

Belly fat rate (g/kg, %) = Belly fat weight (g)/Carcass weight (kg) × 100%.(1)

The length and width of the longissimus thoracis at the thoracolumbar junction were measured with a ruler. The eye muscle area was then calculated using the following formula:

Eye muscle area (cm^2^) = Length (cm) × Width (cm) × 0.7. (2)

At the 45th min and 24th h after slaughtering, pH was measured at the point between the 11th and 12th vertebrae on the left longissimus thoracis (OPTO-STAR, R Mat-thaus, Dresden, German); the Hunter *L*. *a* and *b* value of the longissimus thoracis were determined using a colorimeter (SP60Series, X-Rite Incorporated, Grand Rapids, Michigan, USA) after 45-min and 24-h exposure in the 25 °C, respectively (Pi et al., 2005) [19].

The longissimus thoracis samples were trimmed to a 5 × 2 × 1 cm size, the surface water and fascia were removed and weighed, and the weight was recorded as the initial weight. All the samples were placed in plastic bags filled with air and fastened to avoid evaporation. Then, the bags were vertically hung in the refrigerator at 4 °C. The final weight of the longissimus thoracis samples were determined after 24 and 48 h. The percentage of drip loss was then calculated using the following formula:

Drip loss (%) = (Initial weight − Final weight)/Final weight (g) × 100%.(3)

The longissimus thoracis samples were stored at 4 °C for 24 h. Each 100 g sample was taken and placed in a polyethylene plastic bag. After the air was removed, the bags were sealed and immersed in a water bath at 75 °C for 30 min, and then the bags were cooled for 40 min in 15 °C in running water. The meat samples were taken out from the plastic bags and weighed after wiped off the surface moisture using filter paper. The percentage of cooking loss was then calculated using the following formula:

Cooking loss (%) = (Initial weight − Final weight)/Final weight (g) × 100%.(4)

After the cooking loss was determined, the samples were used to determine shear stress. Along the direction parallel to the muscle fiber, the sample was trimmed into 1 cm (wide) × 1 cm (thick) × 5 cm (long) strips, each strip was cut three times using a muscle tenderness instrument (C-LM3B, Tenovo International Co., Ltd., Beijing, China), and the average value was taken as the shear stress of the samples. Shear direction was perpendicular to the direction of muscle fiber. Drip loss and cooking loss referred to Chao’s experimental methods [20].

The moisture content of the longissimus dorsi muscle was determined by drying at 105 °C, the content of ether extract (EE) was determined by Soxhlet extraction, and the content of crude protein (CP) was determined by automatic Kjeldahl analyzer (K9860, Haineng Instruments Co., Ltd., Jinan, China) and burned at 550 °C for 4 h to determine ash content.

### 2.4. Determination of TVB-N, Lactobacillus and E. coli of Longissimus thoracis 

Approximate 20 g of samples with dimensions of 4 × 4 × 1 cm was taken from the longissimus thoracis. All the samples were stored at 4 °C before further analysis. After 1, 3, 5 and 7 days, the samples were taken out from 4 °C to determine the total volatile basic nitrogen (TVB-N) content. The sample was ground and 10 g subsamples were taken and placed in the distillation tube. After adding 75 mL deionized water, the sample was homogenized to make the sample disperse evenly. One g magnesium oxide was added to the treated sample after 30 min, and immediately connected to the Distiller (K9860, Haineng Instruments Co., Ltd., Jinan, China). The TVB-N content was then calculated using the following formula:

TVB-N (mg/100g) = (V_1_ − V_2_) × 14 × c/m × 100 (5)

where V_1_ is the volume of standard titration solution for sulphuric acid (mL), V_2_ is the volume of standard titration solution for reagent blank digestion of sulphuric acidy (mL), c is the concentration of standard titration solution for sulfuric acid, m is the quality of sample (g), and 100 indicates that the calculation results are converted to milligrams per hundred grams.

For *Lactobacillus* and *E. coli* quantity analysis, the storage and sampling time were the same as above. The composite longissimus sample (1 g) from each pig was diluted with 9 mL of 1% peptone broth and then homogenized. Viable counts of bacteria in the samples were conducted by plating serial 10-fold dilutions (in 1% peptone solution) onto Mac Conkey and LBS agar plates to isolate the *E. coli* and *Lactobacillus*, respectively. The LBS agar plates were incubated for 48 h at 37 °C under anaerobic conditions. The Mac Conkey agar plates were incubated for 24 h at 37 °C. The *E. coli* and *Lactobacillus* colonies were counted immediately after being removed from the incubator.

### 2.5. Data Calculations and Statistical Analysis

All data are presented as the means ± SDs. Data were analyzed by ANOVA of SAS 9.2 (SAS Inst. Inc., Cary, NC, USA) with treatment or storage time as fix effect and individual fattening pig or meat sample from individual pigs as the experimental unit. The significance of the differences among the treatments was tested using Duncan’s multiple range test. Difference was declared to be statistically significant when *p* < 0.05. Tendency was declared with *p* between 0.05 and 0.10.

## 3. Results

### 3.1. Growth Performance

The animal growth performance data are presented in Table 2. Compared with the group SS, group SY, Se-Met and SS + Se-Met had a higher (*p* < 0.05) final weight and ADG, but the effect was more noticeable (*p* < 0.05) in the SS + Se-Met group. Moreover, each group also reduced (*p* < 0.05) the F/G of pigs. However, no difference (*p* > 0.05) was observed in ADFI among the treatments.

### 3.2. Carcass Performance

Results for the carcass performance are shown in Table 3. Adding SY to the diet could increase (*p* < 0.01) the carcass straight length of the fattening pigs. The diets supplemented with SY or SS + Se-Met combination could significantly increase (*p* < 0.01) back fat thickness and skin thickness of the fattening pigs. In addition, adding SY to the diet could significantly increase (*p* < 0.05) the belly fat rate of the fattening pigs. However, there were no (*p* > 0.05) significant effects on dressing percentage, carcass sloping length and eye muscle area of fattening pigs by the addition of different Se sources to the diets.

### 3.3. Meat Quality

The effects of supplementing different Se sources to fattening pigs on the meat quality are presented in Table 4. Compared with the group SS, group SY and SS + Se-Met could significantly reduce (*p* < 0.01) the cooking loss of longissimus thoracis of the fattening pigs. The diet supplemented with Se-Met could significantly reduce (*p* < 0.01) the shearing stress of longissimus thoracis compared with the other treatments. All the treatments had a lower (*p* < 0.01) drip loss of longissimus thoracis 24 h and 48 h after slaughter than the group SS. The diets supplemented with SY and SS + Se-Met could reduce (*P* < 0.01) the *L* value (45 min, 24 h) of longissimus thoracis. All treatments’ b value of longissimus thoracis in 45 min (except for SY) and 24 h (except for SS + Se-Met) was reduced (*p* < 0.01) significantly. However, there were no (*p* > 0.05) differences between the pH (45 min), the pH (24 h), a value (45 min), and a value (24 h) of longissimus thoracis of fattening pigs by adding different Se sources to the diet.

### 3.4. Gross Composition of Longissimus Thoracis

The nutrient contents of longissimus thoracis are given in Table 5. Compared with group SS, group SY and SS + Se-Met could significantly reduce (*p* < 0.05) the moisture content but enhance (*p* < 0.05) the CP content of longissimus thoracis of the fattening pigs. Moreover, the pigs fed with organic Se sources could increase (*p <* 0.01) fat content. However, there were no (*p* > 0.05) significant effects on ASH of longissimus thoracis when the fattening pigs were supplemented with different Se sources.

### 3.5. TVB-N of Longissimus Thoracis 

**Figure 1:**
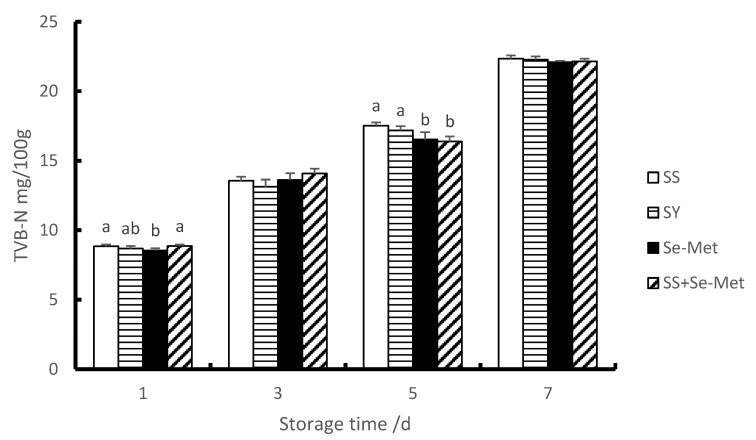
Effects of different Se sources on the TVB-N contents of longissimus thoracis of fattening pigs at different storage time.

The TVB-N value less than 15 mg/100 g is the first level freshness, 15 mg/100 g–20 mg/100 g is the second level freshness, and more than 20 mg/100 g is the deteriorated meat [21,22]. As shown in Figure 1, compared with the first day, the TVB-N contents of each group increased significantly on the third, fifth and seventh days (*p* < 0.01), and there was a trend of further increase. In addition, our results also showed that the contents of TVB-N in the Se-Met group were significantly lower (*p* < 0.05) than those in the SS group on the first day. On the third day, there was no (*p* >0.05) significant difference between each group, but the freshness of longissimus thoracis still belonged to the first grade freshness. On the fifth day, the contents of TVB-N of group Se-Met and group SS + Se-Met were significantly lower (*p* < 0.01) than these in group SS, and the freshness of longissimus thoracis was second-grade fresh. On the seventh day, there was no (*p* < 0.05) significant difference between each group, and the longissimus thoracis of all treatment groups had deteriorated. 

### 3.6. The Lactobacillus Contents of Longissimus Thoracis 

**Figure 2:**
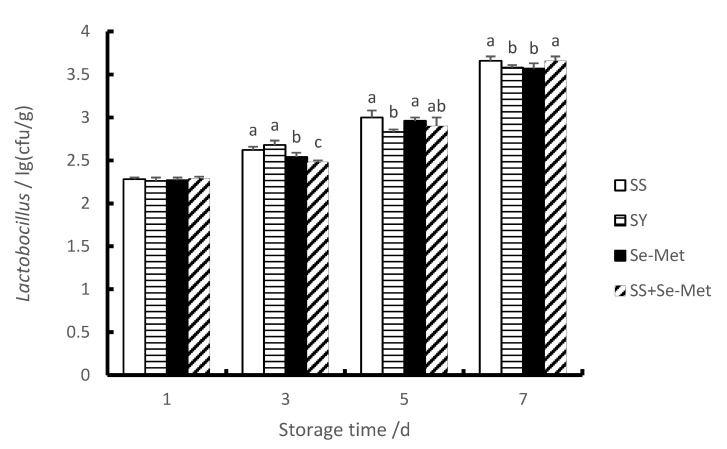
Effects of different Se sources on the *Lactobacillus* contents of longissimus thoracis of fattening pigs at different time periods.

In this study, compared with the first day, the number of *Lactobacilli* in each group increased significantly on the third, fifth and seventh days (*p* < 0.01). As we can see from Figure 2, compared with group SS, there was no (*p* > 0.05) difference in each group on the first day. However, the *Lactobacillus* contents of group Se-Met and group Se-Met + SS were significantly lower (*p* < 0.01) than these in group SS on the third day, and the contents of *Lactobacillus* of group SY were significantly decreased (*p* < 0.05) compared with these in group SS on the fifth days. Moreover, the contents of *Lactobacillus* of group SY and group Se-Met were significantly lower (*p* < 0.05) than that in group SS on the seventh day. Therefore, adding organic Se to the diet could reduce the *Lactobacillus* contents of the longissimus thoracis within the first to the seventh days after slaughter.

### 3.7. The E. coli Contents of Longissimus Thoracis 

**Figure 3:**
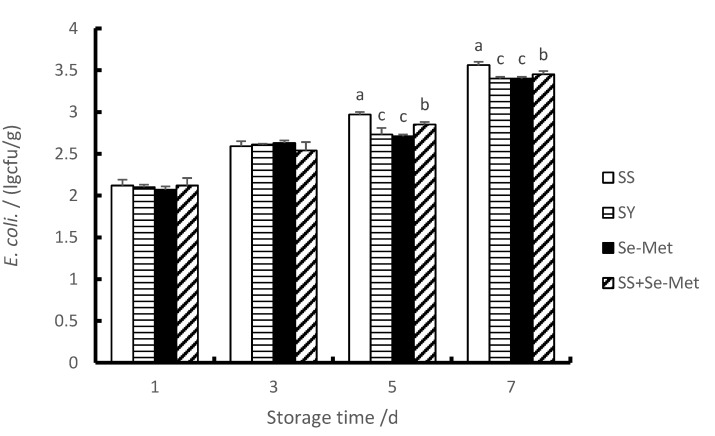
Effects of different Se sources on *E. coli contents* of longissimus thoracis of fattening pigs at different time periods.

In this study, compared with the first day, the number of *E. coli* in each group increased significantly on the third, fifth and seventh days (*p* < 0.01). As we can see from Figure 3, compared with the group SS, the numbers of *E. coli* in the treatments supplemented with organic Se sources (SY or Se-Met) were significantly decreased (*p* < 0.01) on the fifth and seventh day; however, there was no difference (*p* > 0.05) observed between the first and third day. Thus, these results demonstrate that adding organic Se sources to the diet could reduce the *E. coli* contents of the longissimus thoracis after slaughter, which means that the shelf life of pork was extended.

## 4. Discussion

In the present study, the effects of using different Se sources on the meat quality and shelf quality of pork were assessed. Lin et al. (2015) reported that adding 0.5 mg/kg SY significantly increased the weaning weight and ADG of piglets compared with SS group [23]. Zhang et al. (2018) reported that diet supplemented with SY significantly increased ADG of fattening pigs and had an increasing trend of ADFI [24]. The results of this experiment are basically consistent with those of the above studies. In this study, adding organic Se to the diets could significantly improve the ADG and reduce the F/G of pigs in the later stage of fattening; the results were basically consistent with those of our predecessors. However, Jiang et al. (2010) have reported that diet supplemented with SY had no significant effect on ADG, ADFI and F/G of fattening pigs [25], these discrepancies might be due to not only the different production technology of SY, but also the different physical conditions of animals and the stress which animals were being exposed to. 

The weights of pigs are closely related to the indexes of carcass performance. The effects of dietary Se levels and species on carcass performance were different. In this study, diet supplemented with 0.3 mg/kg SY or 0.3 mg/kg SS + Se-Met increased back fat thickness and skin thickness, and adding SY could increase belly fat rate. Wang et al. (2010) reported that adding 0.5% and 1% Se-Met compound additives decreased back fat thickness of fattening pigs [26]. This experiment is inconsistent with the previous results, which possibly because organic Se can not only provide Se sources, but also provide a certain amount of protein, amino acid, vitamin and other nutrients for the body, and promote the accumulation of fat. In this experiment, the addition of organic Se could improve the growth of fattening pigs.

The pH, drip loss, meat color, cooking loss and shear stress are important factors for the meat quality of fattening pigs. After animals are slaughtered, glycogen in muscle will undergo glycolysis, which will produce lactic acid. Increasing lactic acid and H^+^ released from ATP hydrolysis will lead to the decrease of muscle pH [27]. Therefore, pH is one of the most important parameters for meat quality, as it had a positive correlation with the water holding capacity, redness, and tenderness, and a negative correlation with the lightness and drip loss of meat [28,29,30]. In this experiment, the supplementation of different Se sources had no significant effect on the longissimus thoracis pH of the 24 h and 48 h. The above results are consistent with those of Mahan et al. and Yang et al. [13,31]. We speculate that Se would not participate in glycolysis of glycogen in muscle, and the lactate would not be regulated by Se, so the addition of Se had no effect on the pH of longissimus thoracis.

The ability of muscle tissue to retain water is often measured by drip loss. If the value is too high, a large amount of water-soluble protein and sarcoplasmic proteins will be lost, which will seriously affect the color, flavor and tenderness [32]. Zhan et al. (2007) reported that diet supplemented with either 0.30 mg/kg SS or 0.30 mg/kg Se-Met as Se sources could increase the trend of the pH (45 min) value of longissimus thoracis; in particular, the Se-Met group showed better results. Furthermore, drip loss of loin was significantly decreased in the Se-Met group after 8 h and 16 h exposure in a 25 °C room [17]. Studies have shown that an Se-rich diet could improve meat quality, especially protecting muscle from excessive dehydration [33]. In this study, organic Se or combination of organic Se and inorganic Se could significantly reduce the drip loss after 24 h and 48 h. Zou et al. (2005) reported that SY could delay the oxidation of muscle fat, myoglobin and oxymyoglobin, increase the stability of meat color and improve the meat color score, and the meat color value was proportional to the amount of Se added [34]. Jiang et al. (2010) reported that Se-Met could significantly increase a value of the longissimus thoracis of 24 and 48 h, and significantly reduce the *L* value of 12 h, 24 h, 48 h and the b value of 12 h [35]. In this study, adding SY or SS + Se-Met could significantly reduce the *L* value (45 min, 24 h), adding Se-Met or SS + Se-Met could significantly reduce the b value (45 min), but only organic Se could reduce the b value (24 h). In addition, it was also reported that diet supplementation with SS or SY had no effect on the meat color of pigs and sheep [36,37]. Wolter et al. (1999) and Boiago et al. (2014) reported that there was no significant difference in shear force and cooking loss regardless the source or the concentration of Se used [38,39]. However, Khan´s et al. (2018) research showed that adding Se-enriched probiotics could reduce the shearing force of breast muscle and improve muscle tenderness in broiler [40], which is consistent with the results of this experiment. It can be concluded that organic Se had a better effect on improving meat quality than inorganic Se.

Research shows that the content of volatile base nitrogen (TVB-N) is the key to judging meat freshness. Under the joint action of bacteria and endogenous enzymes, the protein in meat is decomposed to produce alkaline volatile substances such as ammonia and amines, which create favorable conditions for bacterial reproduction. With the continuous increase of the number of bacteria, the speed of meat spoilage and deterioration is gradually accelerated, the TVB-N content is also increased, and the shelf life of meat is shortened [21,22,41,42]. The effect of diet Se supplementation on meat quality during different refrigeration periods has been rarely reported. Liu et al. (2010) reported that dietary supplemented with 5% fish oil and 0.3 mg/kg Se had no significant effect on TVB-N of meat for 0–6 days, but had a decreasing trend [43]. Zhao (2017) reported that dietary supplemented with 0.2% or 0.3% Se-enriched germanium yeast reduced TVB-N on the seventh, tenth and fourteenth days of the outer ridge, and on the tenth and fourteenth days of the buttock meat of cattle [44]. In the current study, the TVB-N contents of each treatment increased significantly with the passage of time. However, the longissimus thoracis of organic Se group seems to be fresher than group SS, because the TVB-N contents in the organic Se group were lower those of than group SS on the first and fifth days. but there had no significant effect on the third and seventh days, and meat deteriorated on the seventh day. In this experiment, adding organic Se had the certain effect on reducing TVB-N content of pork. The reason may be that organic Se has the function of scavenging free radicals, slowing down the speed of muscle oxidation, reducing the content of bacteria and endogenous enzymes in pork.

The main spoilage bacteria in chilled meat are *Pseudomonas*, *Enterobacteriaceae*, *Lactobacillus*, *Micrococcus* and *Staphylococcus* [45,46]. *Lactobacillus* is a type of anaerobic or facultative anaerobic Gram-positive bacteria which can ferment sugar and produce lactic acid. In the process of chilled pork storage, *lactobacillus* can decompose sugars to produce lactic acid, which reduces the pH value and the quality of meat [47]. It has also been proved that *Lactobacillus* are the dominant spoilage bacteria in meat [48]. *E. coli* is a kind of aerobic and facultative anaerobic bacterium. Under aerobic conditions, it can produce acid, sulfide and putrescine by fermentation of glucose, amino acid 6-phosphate glucose and alcohol, and has a strong ability of causing corrosion [49]. Preservatives are the main way to inhibit the reproduction of these spoilage bacterium [50]. Yet, chemical preservatives do harm to human body, natural preservatives are needed to replace them [51]. Some studies have shown that dietary Se can enhance the body’s antioxidant capacity, effectively protect the quality of meat during storage, thereby prolonging the shelf life of meat [52]. At present, there are few studies on the effects of Se on the reproduction of microorganisms in meat. Therefore, it is particularly important to study the effects of different Se sources on microbial flora in meat. In this experiment, the number of *Lactobacillus* and *E. coli* in the longissimus thoracis of each treatment increased significantly with the passage of time, which indicated that the freshness of pork is significantly affected by the storage time. Adding different organic Se sources could reduce the number of *Lactobacillus* and *E. coli.* in longissimus thoracis between the third and the seventh days. To a certain extent, it reduced the content of spoilage bacteria in meat. The reason may be that GSH-Px enzymatic reaction can eliminate potentially dangerous hydrogen peroxide and lipid peroxide in animal organisms, thus reducing the reproductive conditions of microbial flora and thus achieving the purpose of inhibiting microbial flora reproduction.

## 5. Conclusions

In conclusion, replacing inorganic Se in diet with organic Se can improve protein and fat content in longissimus thoracis and meat quality of fattening pigs. In addition, organic Se can reduce the TVB-N contents of longissimus thoracis between the third and fifth days, and reduce the microbial contents in longissimus thoracis between the third and seventh days, effectively prevent longissimus thoracis spoilage, and thus prolonging the shelf life of longissimus thoracis. In the current study, the corn-soybean meal diet supplemented with organic Se was an effective strategy to improve meat quality and prolong the shelf life of longissimus thoracis.

## Figures and Tables

**Table 1:** Composition and nutrient contents of basal diet (as feed).

Item	Content
Ingredient, %	
Corn	55.91
Wheat middlings	19
Soybean meal	19
Soybean oil	2.3
CaH_2_PO4	0.4
Limestone	1.3
Sodium chloride	0.4
DL-Met	0.04
L-Lys·HCl	0.6
Thr	0.05
Vitamin-trace mineral premix ^1^	1
Total	100
Analyzed composition	
DE, MJ/kg	13.84
CP, %	16.1
Ca, %	0.62
Available P, %	0.21
Lys, %	1.19
Met, %	0.39
Met + Cys, %	1
Thr, %	0.67
Try, %	0.19
Se, mg/kg	0.34

^1^ Supplied per kilogram of complete diet: 5500 IU vitamin A, 700 IU vitamin D3, 25 IU vitamin E, 2.5 mg vitamin K3, 4.4 mg thiamine, 11 mg riboflavin, 35 mg d-pantothenic acid, 59.5 mg niacin, 330 mg choline, 0.9 mg folic acid, 0.5 mg biotin, 55 µg vitamin B12, 40 mg Mn as manganese sulfate, 130 mg Fe as ferrous sulfate, 130 mg Zn as zinc sulfate, 15 mg Cu as copper sulfate, and 0.35 mg I as calcium iodide.

**Table 2:** Effects of different Se sources on growth performance of fattening pigs ^1^.

Item	SS	SY	Se-Met	SS + Se-Met ^2^	*p*-Value
	0.3 mg/kg	0.3 mg/kg	0.3 mg/kg	0.15 + 0.15 mg/kg	
Initial weight, kg	62.03 ± 3.16	61.45 ± 0.97	61.83 ± 1.25	62.91 ± 2.55	0.810
Final weight, kg	92.80 ^b^ ± 1.29	99.43 ^a^ ± 1.31	99.50 ^a^ ± 3.44	101.68 ^a^ ± 4.31	0.007
ADG, g	615.41 ^b^ ± 18.03	759.46 ^a^ ± 17.01	753.40 ^a^ ± 22.97	775.36 ^a^ ± 36.59	<0.001
ADFI, kg	2.30 ± 0.33	2.44 ± 0.19	2.47 ± 0.11	2.46 ± 0.12	0.644
F/G ^3^	3.72 ^a^ ± 0.16	3.21 ^b^ ± 0.19	3.29 ^b^ ± 0.29	3.17 ^b^ ± 0.02	0.005

^a,b^ Means within a row with different superscripts differ significantly (*p* < 0.05). ^1^ Results are presented as means ± SDs, n = 4. ^2^ SS = sodium selenite; SY = se-enriched yeast; Se-Met = selenomethionine. ^3^ ADG, average daily gain; ADFI, average daily food intake; F/G, gain to feed ratio.

**Table 3:** Effects of different Se sources on carcass performance of fattening pigs ^1^.

Item	SS	SY	Se-Met	SS + Se-Met ^2^	*p*-Value
	0.3 mg/kg	0.3 mg/kg	0.3 mg/kg	0.15 + 0.15 mg/kg	
Dressing percentage, %	72.92 ± 2.11	75.28 ± 1.72	73.16 ± 1.14	72.87 ± 3.15	0.371
Carcass straight length, mm	101.92 ^b^ ± 1.32	104.35 ^a^ ± 1.39	101.50 ^b^± 0.91	101.20 ^b^ ± 0.54	0.006
Carcass sloping length, mm	91.15 ^b^ ± 0.75	92.25 ^a^ ± 0.65	91.45 ^a, b^ ± 0.64	90.75 ^b^ ± 098	<0.086
Back fat thickness, mm	10.63 ^c^ ± 0.19	14.32 ^a^ ± 0.31	10.53 ^c^ ± 0.49	11.91 ^b^ ± 0.68	<0.001
Skin thickness, mm	8.72 ^b^ ± 0.21	9.25 ^a^ ± 0.15	8.56 ^b^ ± 0.22	9.48 ^a^ ± 0.26	<0.001
Belly fat rate, %	10.77 ^b, c^ ± 0.08	12.86 ^a^ ± 0.48	9.99 ^c^ ± 0.40	11.11 ^b, c^ ± 1.09	<0.001
Eye muscle area, cm ^2^	61.86 ± 1.38	63.76 ± 1.90	61.58 ± 1.68	62.46 ± 2.18	0.370

^a–c^ Means within a row with different superscripts differ significantly (*p* < 0.05). ^1^ The results are presented as means ± SD, n = 4. ^2^ SS = sodium selenite; SY = se-enriched yeast; Se-Met = selenomethionine.

**Table 4:** Effects of different Se sources on meat quality of fattening pigs ^1^.

Item	SS	SY	Se-Met	SS + Se-Met ^2^	*p*-Value
	0.3 mg/kg	0.3 mg/kg	0.3 mg/kg	0.15 + 0.15 mg/kg	
pH (45 min)	6.30 ± 0.15	6.17 ± 0.10	6.18 ± 0.12	6.31 ± 0.20	0.414
pH (24 h)	5.89 ± 0.03	5.91 ± 0.08	5.94 ± 0.03	5.72 ± 0.34	0.310
24 h drip loss, %	4.69 ^a^ ± 0.40	3.60 ^b^ ± 0.11	3.30 ^b, c^ ± 0.09	3.23 ^c^ ± 0.09	<0.001
48 h drip loss, %	8.81 ^a^ ± 0.05	7.80 ^b^ ± 0.10	6.98 ^c^ ± 0.13	7.02 ^c^ ± 0.18	<0.001
cooking loss, %	31.99 ^a^ ± 0.69	27.90 ^c^ ± 1.68	31.55 ^a, b^ ± 2.71	29.25 ^b, c^ ± 0.36	0.013
shearing stress, N	33.31 ^a^ ± 0.95	33.27 ^a^ ± 1.34	27.07 ^b^ ± 0.76	32.04 ^a^ ± 1.44	<0.001
*L* value (45 min)	55.20 ^a^ ± 0.34	50.56 ^b^ ± 0.51	54.82 ^a, b^ ± 0.47	54.25 ^b^± 0.79	<0.001
a value (45 min)	9.35 ± 0.13	9.58 ± 0.13	9.60 ± 0.18	9.47± 0.21	0.182
b value (45 min)	9.98 ^a^ ± 0.28	10.13 ^a^ ± 0.33	9.28 ^b^ ± 0.15	9.60 ^b^ ± 0.14	0.001
*L* value (24 h)	57.05 ^a^ ± 0.66	55.48 ^b^ ± 0.75	56.40 ^a^ ± 0.56	51.55 ^c^ ± 0.33	<0.001
a value (24 h)	12.85 ± 0.28	13.15 ± 0.31	13.15 ± 0.21	12.95 ± 0.58	0.602
b value (24 h)	14.18 ^a^ ± 0.17	11.85 ^b^ ± 0.37	12.15 ^b^ ± 0.24	13.88 ^a^ ± 0.22	<0.001

^a–c^ Means within a row with different superscripts differ significantly (*p* < 0.05). ^1^ The results are presented as means ± SD, n = 4. ^2^ SS = sodium selenite; SY = se-enriched yeast; Se-Met = selenomethionine.

**Table 5:** Effects of different Se sources on the gross composition of longissimus thoracis of fattening pigs ^1^.

Item	SS	SY	Se-Met	SS + Se-Met ^2^	*p*-Value
	0.3 mg/kg	0.3 mg/kg	0.3 mg/kg	0.15 + 0.15 mg/kg	
Moisture, %	73.43 ^a^ ± 0.63	72.05 ^b^ ± 0.70	72.74 ^a, b^ ± 0.44	72.22 ^b^ ± 0.67	0.031
ASH, %	1.20 ± 0.01	1.20 ± 0.02	1.20 ± 0.01	1.21 ± 0.01	0.644
CP, %	22.39 ^b^ ± 0.50	23.44 ^a^ ± 0.36	22.75 ^b^ ± 0.19	23.33 ^a^ ± 0.37	0.005
EE, %	2.98 ^b^± 0.07	3.31 ^a^± 0.08	3.31 ^a^ ± 0.07	3.24 ^a^± 0.04	<0.001

^a–b^ Means within a row with different superscripts differ significantly (*p* < 0.05). ^1^ The results are presented as the means ± SD, n = 4. ^2^ SS = sodium selenite; SY = se-enriched yeast; Se-Met = selenomethionine.
